# How is the ecosystem services concept used as a tool to foster collaborative ecosystem governance? A systematic map protocol

**DOI:** 10.1186/s13750-022-00278-8

**Published:** 2022-07-01

**Authors:** Jennifer M. Holzer, Imogen Hobbs, Julia Baird, Gordon Hickey

**Affiliations:** 1 Environmental Sustainability Research Centre, Brock University, 1812 Sir Isaac Brock Way, St. Catharines, ON L2S 3A1 Canada; 2 Department of Natural Resource Sciences, McGill University, 21111 Lakeshore Road, Sainte-Anne-de-Bellevue, QC H9X 3V9 Canada; 3 Department of Geography and Tourism Studies, Brock University, 1812 Sir Isaac Brock Way, St. Catharines, ON L2S 3A1 Canada

**Keywords:** Natural capital, Process, Stakeholder, Boundary object, Management, Governance

## Abstract

**Background:**

While the concept of ecosystem services has been widely adopted by scholars and increasingly used in policy and practice, there has been criticism of its usefulness to decision-makers. This systematic map will collect and analyse literature that frames ES as a collaboration tool, rather than as an ecosystem assessment tool, to answer the research question—*how is the ecosystem services concept used as a tool to foster collaborative ecosystem governance and management?*

**Methods:**

We will search for publications using designated keywords in Web of Science Core Collection, Scopus, grey literature and conservation practitioner databases and websites. The search strategy aims to locate all ecosystem services studies related to collaboration and joint activities. After removing duplicates, we will screen papers in two stages—first by reviewing titles and abstracts and then by reviewing full text. Both stages will screen papers according to the following inclusion criteria: (1) the study is situated in the context of or related to environmental governance or management; (2) the study focuses on ecosystem services being used as a tool for collaboration; (3) the study describes a process resulting from applying the ecosystem services concept as a tool or approach; and (4) the ecosystem services concept is used in the study in a collaboration or group process in a substantial manner. We will exclude papers that do not address the ES concept as a process tool or approach or that use the ecosystem services concept to directly influence specific decisions or policy. Eligible studies will be critically appraised to assess their reporting quality. Studies will then be reviewed to determine: (a) the type of tool or mechanism that is the primary focus or example of the paper, (b) the rationale for using the ES concept, (c) whether a tool or approach was empirically tested in the study, (d) what the study found regarding the usefulness of ES as a tool or approach, and (e) any challenges to their use, if mentioned explicitly. A standard coding spreadsheet will be used by reviewers. Relevant metadata will be extracted for each paper assessed and used to construct an open-access online database. Finally, a narrative synthesis of metadata will be reported based on eligible studies.

**Supplementary Information:**

The online version contains supplementary material available at 10.1186/s13750-022-00278-8.

## Background

The ecosystem services (ES) concept—often defined as the benefits, contributions, or gifts that nature provides to humans—has been widely adopted by scholars and increasingly used in policy and practice documents [1]. Many of the challenges of the ES concept itself and of operationalizing the conceptual, procedural, methodological, and practical aspects of ES have been acknowledged [1, 2]. Early proponents of the concept hoped that it would expand how nature was valued—from being appreciated primarily for resources with obvious market value to highlighting the less marketable and less tangible benefits that nature provides, from pollination services, to soil stabilization to opportunities for recreation [3]. To enhance its feasibility for use in formal decision-making, it became popular to monetize the ES framework, to facilitate comparison of trade-offs. From there, policies and programs were developed to provide payments for ES as well as using ES assessments to inform decision-making generally [4, 5]. However, recent critiques of the dominance of intrinsic and instrumental values in ES scholarship has led to an expansion of these concepts, based largely upon research on cultural ecosystem services. A recent innovation has been the dynamic conversation advocating for greater recognition of *relational values*, “preferences, principles and virtues about human-nature relationships” facilitated by the Intergovernmental Science-Policy Platform on Biodiversity and Ecosystem Services (IPBES) [6].

**Fig. 1:**
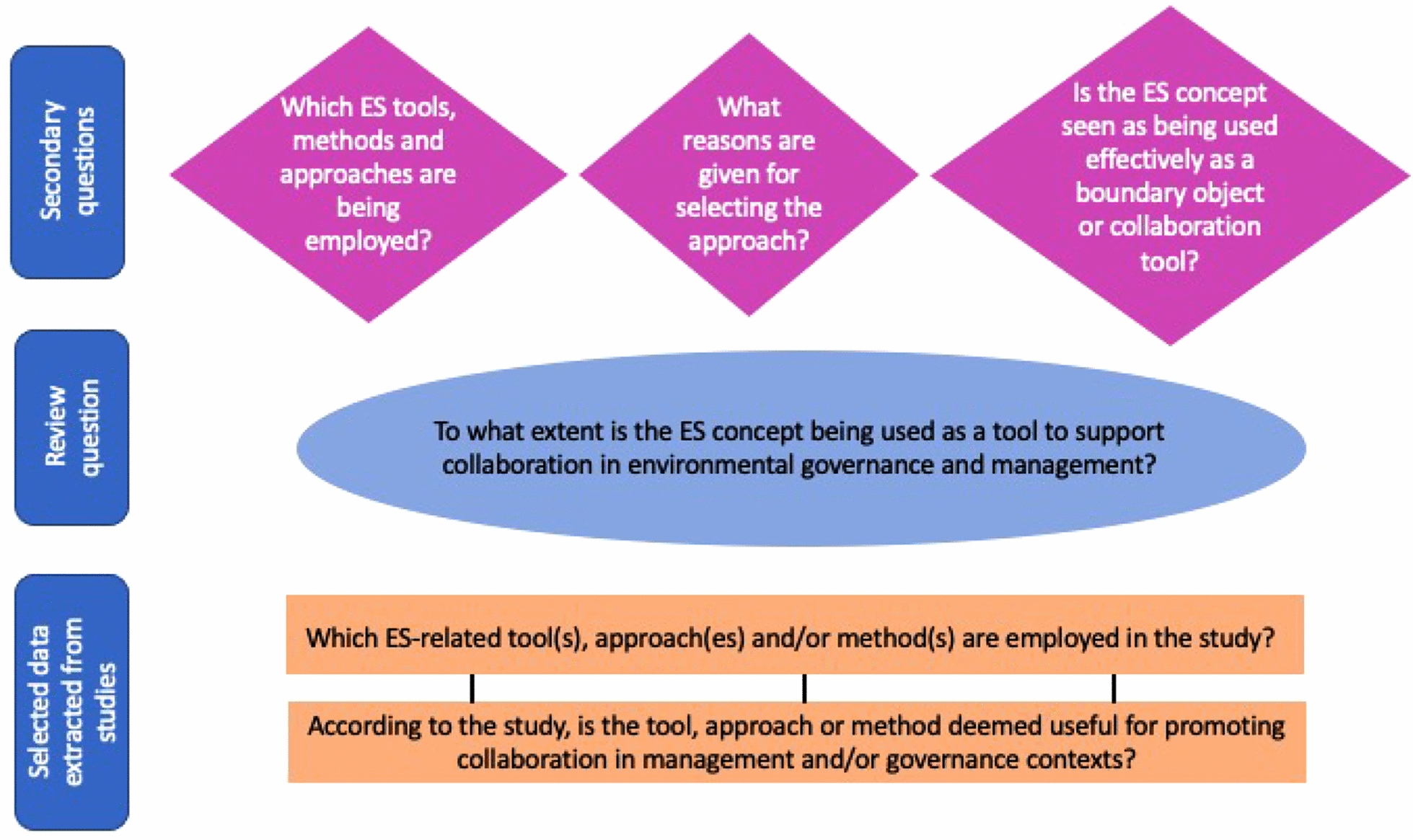
A schematic of conceptual questions motivating the systematic map, core data that will be extracted from the review, and the research question

Critiques of the ES concept have included difficulties standardizing and operationalizing the concept [7, 8], a one-sided perspective (humans taking from nature) [9], and that the concept is situated in the Western scientific paradigm, which may make it problematic for the participation of certain actors such as Indigenous groups [10]. On the other hand, several papers have commented on the usefulness of the ES concept for environmental management highlighting the importance of the ES concept as a tool for advancing collaboration [11–13]. Given the differing perspectives on the ES concept and its usefulness in environmental governance and management, it is important to know to what extent, and how, the ES concept is being used as a tool in collaboration processes. (This is especially salient given IPBES’s (2019) recent emphasis on operationalizing the ES concept at the national and local levels) [14]. This systematic map outlines a process to review academic and practitioner literature to determine to what extent and how the ES concept is being used as a collaboration tool, with direct implications for ecosystem governance and management (Fig. 1).

Recent work reporting on the use of the ES concept as a tool or boundary object led to the need for a systematic map to understand where the balance of evidence lies regarding whether the ES concept is being used as a collaboration tool and how. This is important because strong stakeholder engagement, meaningful participation with diverse groups, and joint knowledge production enable conditions that are known to promote the success of ES projects [15].

### Stakeholder engagement

The authors are affiliated with a Canadian research initiative known as NSERC ResNet, which was established to monitor, model, and manage ES at the landscape and seascape scale, with six designated research hubs and four cross-cutting research teams, focused broadly on the governance, monitoring, and modeling of ES, and knowledge synthesis, respectively. Within that network, our team of social scientists focuses its research on ecosystem governance and management. Because of our de facto role within the scientific network as the team designated to consider the social context of research, along with the network’s decision to employ ES as a core concept, we have, through both formal and informal processes, been privy to many comments and critiques of the ES concept. These comments have come primarily through planning and facilitating workshops with local actors in three of the network’s six designated landscape-based research hubs, as well as in ResNet network meetings. We have heard critiques from researchers across diverse disciplines, as well as from landscape-based actors. This review is an expression of an iterative process of synthesizing these comments and critiques and using them to formulate the above research questions. This is to say that while the stakeholder engagement that contributed to this study was not formal, it was an ongoing process [16], characterized by regular and frequent engagement [17, 18] that helped to identify key issues with using ES as the primary guiding concept in NSERC ResNet and how to move beyond them.

## Objective of the review

The systematic map will address the following primary question: To what extent is the ES concept being used as a tool to support collaboration in environmental governance and management? We seek to understand the extent to which the ES concept has been framed and/or used as a tool to foster collaborative ecosystem governance and management, and to understand how these tools have been conceptualized, deployed in practice, and the benefits and/or shortcomings perceived to be associated with them (see Table 1). By a “tool in a collaboration process,” we mean that the concept was operationalized in a participatory process or exercise that helped to improve understanding of participants’ values or priorities, foster understanding of a landscape or human-nature relationships, or generally advance understanding toward agenda-setting or decision-making (Fig. 1). Examples of using ES as a collaboration tool could be: as a boundary object [13], using maps or mapping activities employing the ES concept [19], or using ES for scenario planning. In general, we are looking for examples of ES tools that advanced collaborative processes rather than those that led directly to formal decisions or policies. The systematic map also aims to answer the following secondary questions: (1) Which ES-related tools, approaches, or methods are being employed? (2) What reasons are given for selecting the approach? (3) Is the ES concept seen as being used effectively as a boundary object or collaboration tool?

**Table 1:** Research question components related to the use of ES as a collaboration tool, inspired by the SPIDER question format (Forming Focused Questions with PICO, https://guides.lib.unc.edu/pico/frameworks) [20]

Setting	Phenomenon of interest	Outcome(s)
Environmental governance and/or management	Ecosystem services as a tool for collaboration	Collaborative processes as a result of application of tools derived from the ecosystem services concept

A few definitions are important here. We define collaboration as “a process in which entities share information, resources, and responsibilities to jointly plan, implement, and evaluate a program of activities to achieve a common goal” (Camarinha-Matos and Afsarmanesh [21], pp. 29). Collaboration encompasses activities like communication and coordination [21]. We are specifically interested in multiparty collaboration, which involves joint decision-making among key stakeholders [22]. Environmental governance refers to a “set of regulatory processes, mechanisms and organizations through which political actors influence environmental actions and outcomes. Governance includes the actions of the state and, in addition, encompasses actors such as communities, businesses, and NGOs” [23]. Environmental management is distinguished from governance in that it represents the products thereof: the resources, plans and activities that are applied as a result of governance [24].

## Methods

The meticulous and transparent methodology of a systematic map make it ideal for addressing the uses, benefits, critiques, and knowledge gaps in the literature. This mapping review will follow the CEE guidelines and standards for evidence synthesis and will conform to the ROSES reporting standards Additional file 1; [25, 26]. The following methodology has been designed to maximize results pertaining to the use of ES as a collaboration tool, identify the knowledge gaps in this field of study, and incorporate several SPIDER components (Table 1; Forming Focused Questions with PICO, https://guides.lib.unc.edu/pico/frameworks), which are used to improve the repeatability and scope of the systematic map protocol [27].

### Searching for articles

We will search abstracts in the Web of Science Core Collection (WoS, using Brock University’s subscription) and Scopus (using McGill University’s subscription) databases for the following search string (“ecosystem service*” OR “nature’s contributions to people” OR “nature’s gift*” OR “natural capital” OR “nature’s benefit*”) AND (collaborat* OR cooperat* OR coordinat* OR joint) AND (manage* OR govern* OR regulat*) AND (tool* or heuristic or metaphor* or interpret*) for all years in WoS and 1974–2022 in Scopus. Search terms were developed by the review team in consultation with a librarian. These search terms aim to capture the diverse uses of ES as a collaboration tool in environmental governance and management. A detailed evolution of this search string can be found in Additional file 2. Fifteen benchmark studies will be used (see Additional file 3); if these articles do not appear in the databases, then the search terms will be adjusted until they do. All searches in this study will be limited to the English language due to the language skills of the review team.

An academic grey literature search will be conducted using the following databases: Proquest Dissertations and Theses Global (using Brock University’s subscription), bioRxiv, the National Science Foundation Award Search database, the Cordis EU Research Results database, and OpenGrey (see Table 2 for complete search strings) [28]. The same search string as above will be used when possible; in several of the grey literature databases search strings needed to be adjusted due to the limitations of the search engines (see Additional file 2 and Tables 2 and 3). For all grey literature searches in all databases, results will be organized by relevance, and only the first 1000 search results will be included for screening [29]. The dates and total number of results will be recorded.

**Table 2:** Listing of academic grey literature databases and search details

Database name	Database contents	Filters	Search strings
Proquest dissertations and theses global	Dissertations and theses	Advanced search; search anywhere except full text; all dates; all source types; all document types; limited to English language results	(“ecosystem service” OR “nature’s contributions to people” OR “nature’s gifts*” OR “natural capital” OR “nature’s benefit*”) AND (collaborat* OR cooperation OR coordination OR joint) AND (manage* OR govern* OR regulat*)
bioRxiv	Biology-related preprints	Advanced search: all dates; all; collections; all sections	“ecosystem service” AND (collaborat* or cooperat* or coordinat* or joint) AND (management OR governance)
National science foundation award search	Articles and grant proposals in non-medical fields of science and engineering	Advanced search keyword field	(1) “ecosystem services” AND (collaborat* or cooperat* or coordinat* or joint) AND (management OR governance); (2) nature* AND gifts; (3) nature* AND “contributions to people”
Cordis EU research results	European Union-funded research projects	Projects and results: all results/select all	“ecosystem service” AND collaborat* AND (management OR governance)
OpenGrey^a^	Grey literature	Basic search	“ecosystem services”

Search strings vary slightly by database due to limitations of advanced search capabilities in each system. We have endeavored to keep searches as consistent as possible

^a^Over the time period which this study was developed, OpenGrey underwent significant changes to its website. The latest search performed (April 25, 2022) accessed the website at https://easy.dans.knaw.nl/ui/datasets/id/easy-dataset

**Table 3:** Listing of practitioner-generated databases and search details

Database name	Database contents	Filters	Search string
Conservation evidence	Practitioner-generated studies	Studies	“ecosystem services” (this term is used in place of a full search string because of the limitations of the database)

In addition to this search of academic grey literature, we will conduct a review of practitioner-generated research. Since many practitioners are not motivated by academic publication, and there may be reasons not to make such research publicly available, reviews must be based on “the best evidence *available* to authors rather than the best evidence [30]” [28]. We have largely followed [28] suggestions for doing a search of practitioner-generated research, using the Conservation Evidence database and searching websites of ecological conservation-related organizations, as described below.

In addition to the above practitioner-generated literature, we will elicit expert knowledge from eight ES scholars to identify non-academic organizations (e.g. conservation organizations like the World Wildlife Fund, quasi-governmental organizations like IPBES, or charitable foundations) that these scholars consider to be the most important producers of studies, reports and/or data related to ES [31]. Specifically, we will show these scholars a list of organizations doing work using the ecosystem services concept and websites obtained through an Advanced Google Search (Additional file 2, Table 2) and ask them if they would consider this list to be complete, or if they would add to or amend the list. This list (Additional file 2, Table 2) was generated by a discussion among the co-authors, supplemented by an Advanced Google Search. The Advanced Google Search used the following search string in the “all these words” field and narrowed results by the English language: (“ecosystem service” OR “nature’s contributions to people” OR “nature’s gifts” OR “nature’s benefits”) AND (collaborat* OR cooperation OR coordination OR joint) AND (management OR governance). We included the first 45 results (after which point the results were saturated by irrelevant documents and websites), with an aim to include only organization websites and reports, so we excluded, for example, articles published in academic journals, press releases, newspaper articles, job announcements, annual reports, and newsletters. We included university department or laboratory websites and excluded websites and reports related to finance and investments. We will finalize the list in a discussion with the eight experts. For websites in the list, we will search the websites for reports and studies related to the topic using the search string (“ecosystem service” OR “nature’s contributions to people” OR “nature’s gifts” OR “natural capital” OR “nature’s benefits”) AND (collaborat* OR cooperation OR coordination OR joint) AND (management OR governance) if possible. If the website does not allow for use of the search string, we will use the keywords: “ecosystem services,” “nature’s contributions to people,” “nature’s gifts,” “natural capital”, “nature’s benefits”, “collaboration”, “management” and “governance”. Reports will be subject to the eligibility criteria detailed below.

## Article screening and study eligibility criteria

### Screening process

The software Covidence will be used to manage the review process, which will occur in two stages. After removing duplicates, titles and abstracts of all papers will be reviewed to exclude those that do not meet the eligibility criteria at this first stage (see “Eligibility criteria” section below for more detail). For the papers that meet the eligibility criteria in the first stage, a second stage screening will follow, where we will skim the full text following the eligibility criteria set out below. Metadata coding will be conducted for the papers that pass the second stage of screening. None of the systematic reviewers have authored articles within the scope of this review, so there is no concern about excluding reviewers from screening their own papers.

The review team will perform a calibration exercise on a random sample of ten studies to ensure that they agree on the inclusion and exclusion criteria [32]. Once the team reaches 90% inter-reliability agreement, two reviewers will independently screen titles and abstracts (stage 1). Disagreements for stage 1 will be resolved by discussion between the two reviewers or by the tie-breaking vote of a third reviewer. Following the completion of title and abstract screening, the team will perform another calibration exercise, with another 10 papers, with the goal of reaching 90% agreement. The full text of studies will then be screened for eligibility, and conflicts for full-text screening will be resolved by a third reviewer [32].

### Eligibility criteria

We will skim papers to determine if they address the following eligibility criteria (derived from research question components per Table 1): (1) settings: study is situated in the context of or related to environmental governance and/or management; (2) phenomena: study has a focus on ecosystem services as a tool for collaboration, (3) outcomes: study describes a collaborative process resulting from applying the ecosystem services concept as a tool or approach. (4) Additional criterion: studies will be eligible for inclusion if they discuss the ES concept being used in some type of collaboration or group process in a *substantial manner*, i.e. this includes empirical studies, both those using experiments or games as well as those using real-world cases. Only English-language studies will be eligible, due to the knowledge constraints of the reviewers. We have defined ‘substantial manner’ to mean that the paper mentions that either the objective or result of using the ES concept was to affect the nature of interactions among members of a particular group. If the ES concept is used in a more results-focused way, such as an analytical framework (e.g. ES assessments) or policy tool (e.g. payments for ecosystem services), the paper will be excluded. A grey area would be, for example, papers that discuss workshops held with stakeholders for the purpose of ranking or discussing ES. If such a paper discusses the quality of the interactions among the stakeholders substantially (more explanation than a single sentence), they will be included. Excluded articles will be included in a list (including reasons for exclusion) that will be available as supplementary information. Additional file 4 contains the framework used to screen, exclude, and check reporting quality of studies; an example is provided as Additional file 5, and screening criteria are diagrammed as a coding tree in Additional file 6. Any articles with missing data will be noted; however, due to the nature of the evidence under review, missing data will not be considered a critical issue and not necessarily necessitate exclusion from the review.

### Critical appraisal of study validity assessment

We will not be conducting a study validity assessment, which is in line with accepted methodological guidance for systematic maps [29], instead, we will extract information about reporting quality. The reporting quality of each paper will be rated as ‘red flag’, ‘green flag,’ or ‘undetermined’. Assessment will be conducted using the following criteria for papers included in the review:

1. The paper contains a complete and detailed description of methods.
2. The paper makes data available.
3. The paper draws conclusions using suitable data.
4. There are no red flags that would be of clear concern to an academic peer reviewer, such as personal conflicts of interest, private sector funders of the study, etc.

Any paper that does not pass the test of exemplifying these four criteria will be labeled with a ‘red flag.’ These ‘red flag’ papers will be discussed between the two reviewers, and if the second reviewer agrees with the first reviewer’s assessment, then the study will be marked with a ‘red flag’ in the dataset. This will not cause it to be excluded from the study but is simply so that a note is made and these notes are mentioned in the study synthesis and presentation. Studies will be labeled ‘undetermined’ if the reviewers deem that they cannot determine whether the study passes the three criteria above. Grey and practitioner literature will also be critically assessed for reporting quality in this way.

### Data coding strategy

Relevant metadata consist of key information that will be extracted from each included study where ES was used as a tool to foster collaborative governance and management. Our data coding strategy is based on a similar strategy created by Lemasson et al. [33]. Metadata will be coded for each respective study that has passed screening requirements and will include a unique identifier. Studies will be coded by their bibliographic information (article reference, year, and publication journal) and reviewer information; the full framework and an example are available in Additional Files 4 and 5 respectively. These studies and extracted metadata will be used to create an open-access database. If data are missing, we will write to the corresponding author (or if no corresponding author is indicated, then the first author) of the paper to obtain or confirm missing or unclear data.

The repeatability of the data coding process will be tested by both reviewers independently entering metadata for 10 papers and then checking for discrepancies. Any discrepancies will be discussed to determine how to code, and a third reviewer brought in to resolve disagreements.

The included papers will be reviewed and the following data will be extracted from them (i.e., “core questions”):

1. What tool/mechanism is the focus or primary focus or example in this paper?
2. Why was this tool chosen for this study?
3. How was this tool empirically tested in a collaborative process (case study, experiment, etc.)?
4. What did the study find regarding the usefulness of ES as a tool in a group process?
5. If clearly stated in the paper, what challenges, if any, were mentioned about the process of implementing the tool in a collaborative process?

These data will be tracked by reviewers in a common spreadsheet. Papers will be coded inductively and grouped by type of tool or mechanism in order to understand author objectives and conclusions about utilizing each type of tool. Our data coding framework is available as Additional file 7.

### Study mapping and presentation

The findings and respective metadata from this study will be available through *Environmental Evidence* in an open-access database and as a systematic map. Descriptive statistics (frequencies, mode, range) may be used to describe the nature of the body of literature reviewed (i.e. the metadata). The data collected that address the core questions of interest will be coded using an inductive, in vivo approach [34] and presented as themes emerging in response to the core questions above. A narrative synthesis of metadata will be conducted from eligible studies [35], and figures or tables will be used to present the results as the need arises. Figures will be particularly useful for highlighting knowledge gaps and knowledge clusters, as described in Lemasson et al. [33]. Knowledge gaps will be identified where one or more of the core questions of interest cannot be (fully) answered from the available evidence from the systematic mapping review. These findings can be used to inform decision-makers and spawn further research into the potential uses of the ES concept as a collaboration tool through identification of knowledge gaps. Specifically, this protocol could be used to address systematic review questions such as “*How does the ES concept affect decision-makers’ values and priorities in environmental governance*,” and “*How does the ES concept relate to human-nature relationships?*”.

## Supplementary Information

**Additional file 1.** ROSES protocol checklist.

**Additional file 2.** This spreadsheet contains the evolution of the search string. All search string changes for Scopus, Web of Science, and Grey literature are viewable here.

**Additional file 3.** Benchmark papers.

**Additional file 4.** This spreadsheet contains the skeleton framework used to screen, exclude, and appraise reporting quality of studies.

**Additional file 5.** This document contains a full example of eligibility screening framework.pdf.

**Additional file 6.** This document contains the Coding Tree used to screen studies. It follows the same framework outlined in Additional File 4.

**Additional file 7.** Metadata coding framework.

## Data Availability

The datasets used and/or analysed during the current study are available from the corresponding author on reasonable request.

## References

[1] Jax K, Furman E, Saarikoski H, Barton DN, Delbaere B, Dick J, et al. Handling a messy world: lessons learned when trying to make the ecosystem services concept operational. Ecosyst Serv. 2018;29:415–27. 10.1016/j.ecoser.2017.08.001

[2] Winkler KJ, Dade MC, Rieb JT. Mismatches in the ecosystem services literature—a review of spatial, temporal, and functional–conceptual mismatches. Curr Landscape Ecol Rep. 2021;6(2):23–34. 10.1007/s40823-021-00063-2

[3] Raymond CM, Singh GG, Benessaiah K, Bernhardt JR, Levine J, Nelson H, et al. Ecosystem services and beyond: using multiple metaphors to understand human–environment relationships. Bioscience. 2013;63(7):536–46. 10.1525/bio.2013.63.7.7

[4] Braat LC, de Groot R. The ecosystem services agenda: bridging the worlds of natural science and economics, conservation and development, and public and private policy. Ecosyst Serv. 2012;1(1):4–15. 10.1016/j.ecoser.2012.07.011

[5] Gómez-Baggethun E, de Groot R, Lomas PL, Montes C. The history of ecosystem services in economic theory and practice: from early notions to markets and payment schemes. Ecol Econ. 2010;69(6):1209–18. 10.1016/j.ecolecon.2009.11.007

[6] Chan KM, Gould RK, Pascual U. Editorial overview: Relational values: what are they, and what’s the fuss about? Curr Opin Environ Sustain. 2018;35:A1-7. 10.1016/j.cosust.2018.11.003

[7] Lautenbach S, Mupepele A-C, Dormann CF, Lee H, Schmidt S, Scholte SSK, et al. Blind spots in ecosystem services research and challenges for implementation. Reg Environ Change. 2019;19(8):2151–72. 10.1007/s10113-018-1457-9

[8] Polasky S, Tallis H, Reyers B. Setting the bar: standards for ecosystem services. Proc Natl Acad Sci USA. 2015;112(24):7356–61. 26082540 10.1073/pnas.1406490112 PMC4475943

[9] Comberti C, Thornton TF, de Echeverria VW, Patterson T. Ecosystem services or services to ecosystems? Valuing cultivation and reciprocal relationships between humans and ecosystems. Glob Environ Change. 2015;34, 247–62. 10.1016/j.gloenvcha.2015.07.007

[10] Von der Porten S, De Loë R, Plummer R. Collaborative environmental governance and indigenous peoples: recommendations for practice. Environ Pract. 2015;17(2):134–44. 10.1017/S146604661500006X

[11] Abson DJ, von Wehrden H, Baumgärtner S, Fischer J, Hanspach J, Härdtle W, et al. Ecosystem services as a boundary object for sustainability. Ecol Econ. 2014;103:29–37. 10.1016/j.ecolecon.2014.04.012

[12] Grêt-Regamey A, Sirén E, Brunner SH, Weibel B. Review of decision support tools to operationalize the ecosystem services concept. Ecosyst Serv. 2017;26:306–15. 10.1016/j.ecoser.2016.10.012

[13] Steger C, Hirsch S, Evers C, Branoff B, Petrova M, Nielsen-Pincus M, et al. Ecosystem services as boundary objects for transdisciplinary collaboration. Ecol Econ. 2018;143:153–60. 10.1016/j.ecolecon.2017.07.016

[14] Intergovernmental science-policy platform on biodiversity and ecosystem services. Summary for policymakers of the global assessment report on biodiversity and ecosystem services. Zenodo. 2019 https://zenodo.org/record/3553579. Accessed Feb 8 2022.

[15] Posner SM, McKenzie E, Ricketts TH. Policy impacts of ecosystem services knowledge. Proc Natl Acad Sci USA. 2016;113(7):1760–5. 26831101 10.1073/pnas.1502452113 PMC4763745

[16] Keown K, Van Eerd D, Irvin E. Stakeholder engagement opportunities in systematic reviews: knowledge transfer for policy and practice. J Contin Educ Heal Prof. 2008;28(2):67–72. 10.1002/chp.159 18521874

[17] Camilleri MA. Valuing stakeholder engagement and sustainability reporting. Corp Reputation Rev. 2015;18(3):210–22. 10.1057/crr.2015.9

[18] Lockwood M, Davidson J, Curtis A, Stratford E, Griffith R. Multi-level environmental governance: lessons from Australian natural resource management. Aust Geogr. 2009;40(2):169–86. 10.1080/00049180902964926

[19] Reilly K, Adamowski J, John K. Participatory mapping of ecosystem services to understand stakeholders’ perceptions of the future of the Mactaquac dam. Canada Ecosystem Services. 2018;30:107–23. 10.1016/j.ecoser.2018.01.002

[20] LibGuides: Forming focused questions with Pico: Other Question frameworks [Internet]. Other Question Frameworks - Forming Focused Questions with PICO - LibGuides at University of North Carolina at Chapel Hill. https://guides.lib.unc.edu/pico/frameworks Accessed Feb 8 2022.

[21] Camarinha-Matos LM, Afsarmanesh H. On reference models for collaborative networked organizations. Int J Prod Res. 2008;46(9):2453–69. 10.1080/00207540701737666

[22] Feist A, Plummer R, Baird J, Mitchell SJ. Examining collaborative processes for climate change adaptation in new Brunswick. Canada Environ Manage. 2020;65(5):665–77. 32215695 10.1007/s00267-020-01284-7

[23] Lemos MC, Agrawal A. Environmental governance. Annu Rev Environ Resour. 2006;31(1):297–325. 10.1146/annurev.energy.31.042605.135621

[24] Lockwood M. Good governance for terrestrial protected areas: a framework, principles and performance outcomes. J Environ Manage. 2010;91(3):754–66. 19896262 10.1016/j.jenvman.2009.10.005

[25] Collaboration for Environmental Evidence. Guidelines and standards for evidence synthesis in environmental management. Version 5.0 .In: AS Pullin, GK Frampton, B Livoreil, G Petrokofsky, eds. 2018. www.environmentalevidence.org/information-for-authors. Accessed 5 Apr 2022

[26] Haddaway NR, Macura B, Whaley P, Pullin AS. ROSES RepOrting standards for Systematic Evidence Syntheses: pro forma, flow-diagram and descriptive summary of the plan and conduct of environmental systematic reviews and systematic maps. Environ Evid. 2018;7(1):1–8. 10.1186/s13750-018-0121-7

[27] Berger-Tal O, Greggor AL, Macura B, Adams CA, Blumenthal A, Bouskila A, et al. Systematic reviews and maps as tools for applying behavioral ecology to management and policy. Behav Ecol. 2019;30(1):1–8. 10.1093/beheco/ary130

[28] Haddaway NR, Bayliss HR. Shades of grey: two forms of grey literature important for reviews in conservation. Biol Cons. 2015;191:827–9. 10.1016/j.biocon.2015.08.018

[29] James KL, Randall NP, Haddaway NRA. methodology for systematic mapping in environmental sciences. Environ Evid. 2016;5(1):1–13. 10.1186/s13750-016-0059-6

[30] Pullin AS, Salafsky N. Save the whales? Save the rainforest? Save the data!: editorial. Conserv Biol. 2010;24(4):915–7. 20636614 10.1111/j.1523-1739.2010.01537.x

[31] Hemming V, Burgman MA, Hanea AM, McBride MF, Wintle BC. A practical guide to structured expert elicitation using the IDEA protocol. Methods Ecol Evol. 2018;9(1):169–80. 10.1111/2041-210X.12857

[32] Ayala AP, Sikora L, Kirtley S, Labelle PR. Barriers and facilitators for early career researchers completing systematic or scoping reviews in health sciences: a scoping review. OSF Preprints. 2019. 10.31219/osf.io/gkzf2.

[33] Lemasson AJ, Knights AM, Thompson M, Lessin G, Beaumont N, Pascoe C, et al. Evidence for the effects of decommissioning man-made structures on marine ecosystems globally: a systematic map protocol. Environ Evid. 2021;10(1):4. 10.1186/s13750-021-00218-y

[34] Saldaña J. The coding manual for qualitative researchers. 2nd ed. Los Angeles: SAGE; 2013. p. 303.

[35] Snilstveit B, Oliver S, Vojtkova M. Narrative approaches to systematic review and synthesis of evidence for international development policy and practice. J Dev Eff. 2012;4(3):409–29. 10.1080/19439342.2012.710641

