# Supplementary material for: How is the ecosystem services concept used as a tool to foster collaborative ecosystem governance? A systematic map protocol
Source: Environ Evid. 2022 Jul 1;11:25. doi: 10.1186/s13750-022-00278-8 (PMC11378854; doi:10.1186/s13750-022-00278-8)
Supplement: Supplementary file 3 — Additional file 3. Benchmark papers. [file 13750_2022_278_MOESM3_ESM.pdf]

ReadMe

Additional File 3.pdf: Benchmark papers

Allen K, Castellano C, Pessagno S. Using dialogue to contextualize culture, ecosystem services, and cultural ecosystem services. Ecology and Society. 2021 Apr 26;26(2); doi/10.5751/ES-12187-260207

Armatas CA, Borrie WT, Watson AE. A Social Science Method for Public Engagement in the Context of Natural Resource Planning in the United States. Society & Natural Resources. 2021 Dec 13:1-21.; doi/10.1080/08941920.2021.1996666

Carmen E, Watt A, Carvalho L, Dick J, Fazey I, Garcia-Blanco G, Grizzetti B, Hauck J, Izakovicova Z, Kopperoinen L, Liquete C. Knowledge needs for the operationalisation of the concept of ecosystem services. Ecosystem Services. 2018 Feb 1;29:441-51.; doi/10.1016/j.ecoser.2017.10.012

De Vreese R, Leys M, Dendoncker N, Van Herzele A, Fontaine CM. Images of nature as a boundary object in social and integrated ecosystem services assessments. Reflections from a Belgian case study. Ecosystem Services. 2016 Dec 1;22:269-79.; doi/10.1016/j.ecoser.2016.06.008

Fletcher PJ, Kelble CR, Nuttle WK, Kiker GA. Using the integrated ecosystem assessment framework to build consensus and transfer information to managers. Ecological Indicators. 2014 Sep 1;44:11-25.; doi/10.1016/j.ecolind.2014.03.024

Hayek UW, Teich M, Klein TM, Grêt-Regamey A. Bringing ecosystem services indicators into spatial planning practice: Lessons from collaborative development of a web-based visualization platform. Ecological Indicators. 2016 Feb 1;61:90-9.; doi/10.1016/j.ecolind.2015.03.035

Lopes R, Videira N. A collaborative approach for scoping ecosystem services with stakeholders: the case of Arrabida Natural Park. Environmental Management. 2016 Aug;58(2):323-42.; doi/10.1007/s00267-016-0711-5

McKenzie E, Posner S, Tillmann P, Bernhardt JR, Howard K, Rosenthal A. Understanding the use of ecosystem service knowledge in decision making: lessons from international experiences of spatial planning. Environment and Planning C: Government and Policy. 2014 Apr;32(2):320-40.; doi/10.1068/c12292j

Moreau C, Barnaud C, Mathevet R. Conciliate agriculture with landscape and biodiversity conservation: a role-playing game to explore trade-offs among ecosystem services through social learning. Sustainability. 2019 Jan;11(2):310.**;** doi/10.3390/su11020310

Moreno J, Palomo I, Escalera J, Martín-López B, Montes C. Incorporating ecosystem services into ecosystem-based management to deal with complexity: a participative mental model approach. Landscape Ecology. 2014 Oct;29(8):1407-21.; doi/10.1007/s10980-014-0053-8

Palomo I, Martín-López B, López-Santiago C, Montes C. Participatory scenario planning for protected areas management under the ecosystem services framework: the Doñana social-ecological system in southwestern Spain. Ecology and Society. 2011 Mar 1;16(1).

Plant R, Prior T. An ecosystem services framework to support statutory water allocation planning in Australia. International journal of river basin management. 2014 Jul 3;12(3):219-30.; doi/10.1080/15715124.2013.865635

Primmer E, Furman E. Operationalising ecosystem service approaches for governance: do measuring, mapping and valuing integrate sector-specific knowledge systems?. Ecosystem Services. 2012 Jul 1;1(1):85-92.; doi/10.1016/j.ecoser.2012.07.008

Segura M, Maroto C, Belton V, Ginestar C. A new collaborative methodology for assessment and management of ecosystem services. Forests. 2015 May;6(5):1696-720.; doi/10.3390/f6051696

Steger C, Hirsch S, Evers C, Branoff B, Petrova M, Nielsen-Pincus M, Wardropper C, Van Riper CJ. Ecosystem services as boundary objects for transdisciplinary collaboration. Ecological economics. 2018 Jan 1;143:153-60.; doi/10.1016/j.ecolecon.2017.07.016
