# Supplementary material for: How is the ecosystem services concept used as a tool to foster collaborative ecosystem governance? A systematic map protocol
Source: Environ Evid. 2022 Jul 1;11:25. doi: 10.1186/s13750-022-00278-8 (PMC11378854; doi:10.1186/s13750-022-00278-8)
Supplement: Supplementary file 5 — Additional file 5. This document contains a full example of eligibility screening framework.pdf. [file 13750_2022_278_MOESM5_ESM.docx]

Readme:

This document contains a full example of the eligibility screening framework outlined in Additional File 4.pdf

| **Category** | Journal Article |
| --- | --- |
| **Study ID** | 398 |
| **Person Screening** | ILH |
| **Screening Date** | March 23 |
| **Study Reference** | Alonso ML, Gutiérrez MR. Biodiversity, ecosystem services and teaching: do our students understand how the functioning of ecosystems contributes to human well-being?. Limnetica. 2017;36(2):479-90. |
| **Authors** | Alonso ML, Gutiérrez MR. |
| **Year** | 2017 |
| **Title** | Biodiversity, ecosystem services and teaching: do our students understand how the functioning of ecosystems contributes to human well-being? |
| **Abstract** | The ecosystem services approach is being used by academics, researchers and managers to support and inform environmental management and biodiversity conservation. However, including this concept in training programs for university students is still poor or nonexistent. We analyzed student preferences in the degrees of Biology and Environmental Sciences at the University of Murcia (east Spain) of the ecosystem services delivered by two ecosystem types (aquatic and arid ecosystems). We specifically explored: What categories of ecosystem services (provisioning, regulating and cultural) is preferably selected by students in the two analyzed ecosystem types? Do students select different ecosystem services in each analyzed ecosystem?; Is this selection conditioned by gender?; Are students able to recognize the ecosystem services that arise from ecosystem functioning?. A survey was completed by 264 students. It was designed to assess the perception of ecosystem services of two ecosystems in the Murcia Region: the Segura River and an arid landscape. Before completing the questionnaire, students were provided with a brief explanation about the concept and typology of the ecosystem services. Each student selected the five most important ecosystem services in each ecosystem of the 22 proposed. Provisioning services were preferentially selected by students for both ecosystems. Regulating arid ecosystem services, were selected by less than half the students. No significant differences were found in the selection of ecosystem services provided by the two ecosystems between males and females, although most regulating services provided by the arid ecosystem were preferentially selected by females. For the aquatic ecosystem, “freshwater” was selected as the main provisioning service. For the arid ecosystem, students preferentially selected “mineral raw materials” and all the regulating services were selected by less than 20% of students. A slight overlap was noted between the services selected by students and was proposed as being “very important” by ecosystem experts. We propose incorporating the framework of sustainability sciences as a platform to teach complex and interdisciplinary issues, the use of new pedagogical methods and the collaborative participation of university teachers. |
| **Source** | Limnetica |
| **Source Type** | Academic Journal |
| **Person Extracting** | ILH |
| **Stage 1 Screening (Title and Abstract) - (1) Setting: Is the study situated in the context of or related to environmental governance and/or management?** | No |
| **Stage 1 Screening (Title and Abstract) - (2) Phenomena: Is there a focus on ecosystem services as a tool for collaboration?** | No |
| **Stage 1 Screening (Title and Abstract) -(3) Outcomes: Does the study describe a process resulting from applying the ecosystem services concept as a tool or approach?** | Yes |
| **Stage 1 Screening (Title and Abstract) - (4) Additional criterion: Is the ES concept being used in a collaboration or group process in a substantial manner?** | No |
| **Included or excluded at end of Stage 1?** | Excluded |
| **Stage 2 Screening (Full text) - (1) Setting: Is the study situated in the context of or related to environmental governance and/or management?** | N/A |
| **Stage 2 Screening (Full text) - (2) Phenomena: Is there a focus on ecosystem services as a tool for collaboration?** | N/A |
| **Stage 2 Screening (Full text) - (3) Outcomes: Does the study describe a process resulting from applying the ecosystem services concept as a tool or approach?** | N/A |
| **Stage 2 (Full text) - (4) Additional criterion: Is the ES concept being used in a collaboration or group process in a substantial manner?** | N/A |
| **Included or excluded at end of Stage 2?** | Excluded |
| **If excluded, reason for exclusion** | Does not meet Stage 1 criteria 1, 2 or 4 |
| **Critical appraisal of reporting quality (Red flag, green flag, undetermined)** | N/A |
