# Supplementary figures and images for: How is the ecosystem services concept used as a tool to foster collaborative ecosystem governance? A systematic map protocol
Source: Environ Evid. 2022 Jul 1;11:25. doi: 10.1186/s13750-022-00278-8 (PMC11378854; doi:10.1186/s13750-022-00278-8)

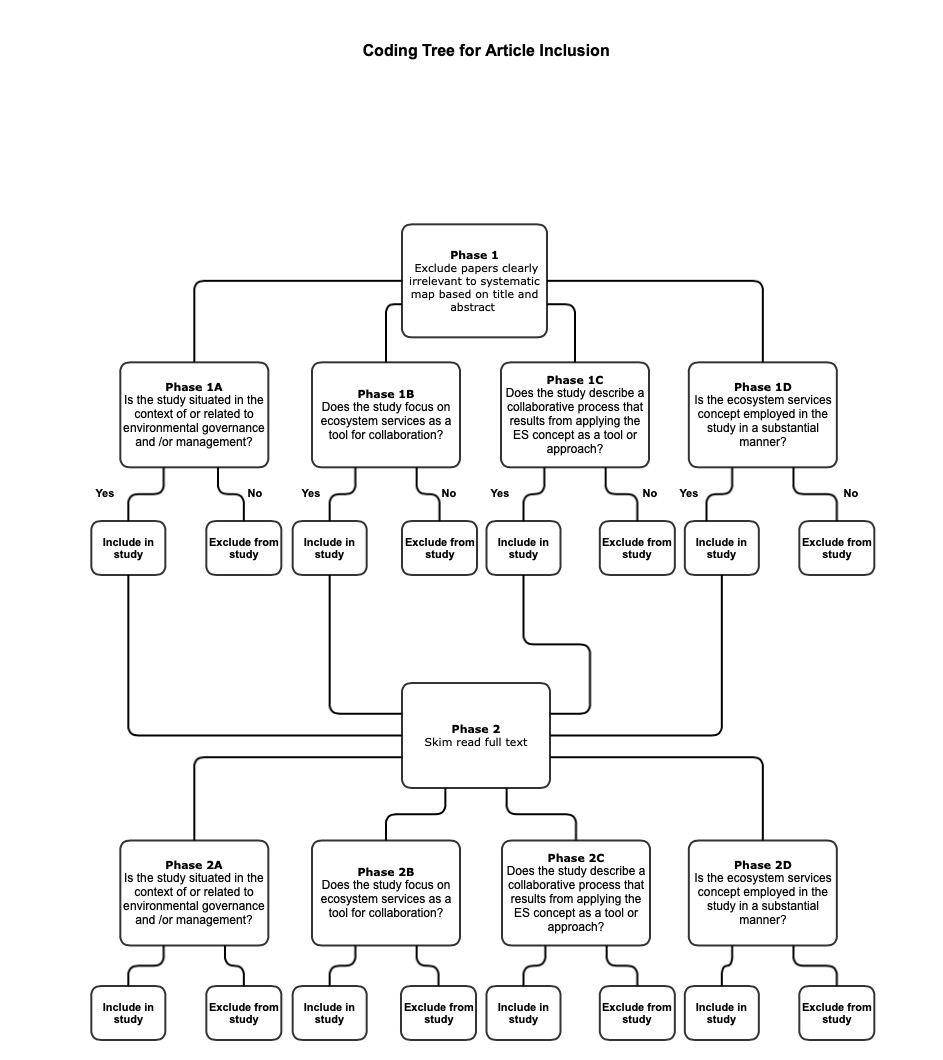

Supplement: Supplementary file 6 — Additional file 6. This document contains the Coding Tree used to screen studies. It follows the same framework outlined in Additional File 4. [file 13750_2022_278_MOESM6_ESM.jpg]
